# An NF-Y-Dependent Switch of Positive and Negative Histone Methyl Marks on CCAAT Promoters

**DOI:** 10.1371/journal.pone.0002066

**Published:** 2008-04-30

**Authors:** Giacomo Donati, Raffaella Gatta, Diletta Dolfini, Andrea Fossati, Michele Ceribelli, Roberto Mantovani

**Affiliations:** Dipartimento di Scienze Biomolecolari e Biotecnologie, Università di Milano, Milano, Italy; University of Munich and Center of Integrated Protein Science, Germany

## Abstract

**Background:**

Histone tails have a plethora of different post-translational modifications, which are located differently in “open” and “closed” parts of genomes. H3K4me3/H3K79me2 and H4K20me3 are among the histone marks associated with the early establishment of active and inactive chromatin, respectively. One of the most widespread promoter elements is the CCAAT box, bound by the NF-Y trimer. Two of NF-Y subunits have an H2A-H2B-like structure.

**Principal findings:**

We established the causal relationship between NF-Y binding and positioning of methyl marks, by ChIP analysis of mouse and human cells infected with a dominant negative NF-YA: a parallel decrease in NF-Y binding, H3K4me3, H3K79me2 and transcription was observed in promoters that are dependent upon NF-Y. On the contrary, changes in the levels of H3K9-14ac were more subtle. Components of the H3K4 methylating MLL complex are not recruited in the absence of NF-Y. As for repressed promoters, NF-Y removal leads to a decrease in the H4K20me3 mark and deposition of H3K4me3.

**Conclusions:**

Two relevant findings are reported: (i) NF-Y gains access to its genomic locations independently from the presence of methyl histone marks, either positive or negative; (ii) NF-Y binding has profound positive or negative consequences on the deposition of histone methyl marks. Therefore NF-Y is a fundamental switch at the heart of decision between gene activation and repression in CCAAT regulated genes.

## Introduction

Specific histone post-translational modifications are known marks of peculiar chromatin environments. Some of them are associated with accessible, active chromatin, others with heterochromatin, either constitutive or facultative [1], [2]. Specifically, H3K4me3 and H3K79me2 are found in regions that are transcribed, or poised to rapid induction [3]. Their presence *in vivo* has been detailed at the single gene level and genome-wide analysis confirmed their widespread distribution in the proximity of promoters [4]–[8]. These H3 methylations are brought in by MLL and Dot1 complexes [9]–12. In addition to positive modifications, histone tails carry tri-methylations associated to inactivate chromatin, with H3K9, H3K27 and H4K20 being the most studied so far [13]. Specifically, H4K20me3 is the result of the activity of Suv4-20, is associated to heterochromatin [14] and is important for cell-cycle progression [15].

Selective deposition of methyl marks is exerted through different mechanisms. For positive marks, this includes phosphorylation of PolII [16], [17] and promoter recruitment of hBRE1 and MLL complexes by sequence-specific transcription factors -TF- such as p53 [18]. However, removal of another TF, MYC, apparently always associated to H3K4me3 and H3K79me2, leaves these marks intact, suggesting that they are important for MYC to find its targets *in vivo* [19]. This indicates that TFs could behave differently in terms of recruitment of methylating complexes. These findings are paralleled for negative marks by the notion that sequence-specific TFs recruit Polycomb complexes to specific locations [20].

The CCAAT box is one of the most frequent promoter elements, being found in 60–70% of them by bioinformatic analysis [21]–[23] and ChIP on chip experiments [24]. The position of CCAAT boxes within promoters is relatively fixed, at −60/−100 from the Transcriptional Start Site, TSS [25; D. Dolfini R.M., in preparation]. Whenever tested, the element significantly contributes to promoter activity. The CCAAT activator is NF-Y, an ubiquitous trimer composed of NF-YA, NF-YB and NF-YC, all necessary for DNA binding [26]. NF-YB and NF-YC have conserved histone fold motifs resembling H2B-H2A and their heterodimerization is essential for NF-YA association [27]. NF-Y is specifically required for genes regulated during the cell-cycle, and inducible by external stimuli: essentially all G2/M genes, for example, are dependent upon NF-Y [21], [28]. NF-Y is generally important to recruit neighbouring TFs and, in some systems, PolII, before induction of transcription [29]. Consistent with the widespread activity, inactivation of the CBF-B/NF-YA gene in mice is embryonic lethal at a very early stage of development [30].

The relationship between NF-Y binding and histone marks has been investigated in ER-stress inducible genes: NF-Y was present with H3K4me3 and H3K79me2 in basal conditions on all promoters tested [31], [32]. Interestingly, correlative ChIP on chip experiments on tiling arrays coupled to expression analysis identified a distinct cohort of NF-Y sites that are devoid of H3K4me3, suggesting that this modification is not required for NF-Y access to CCAAT boxes. Some of these sites had H3K27me3, and all were positive for H4K20me3. Furthermore, removal of NF-Y leads to an increase in transcription. These findings prompted us to investigate the relationships between NF-Y binding and methylation marks on CCAAT promoters in cause-effect experiments.

## Results

### NF-Y is required for transcription of active CCAAT promoters

**Figure 1:**
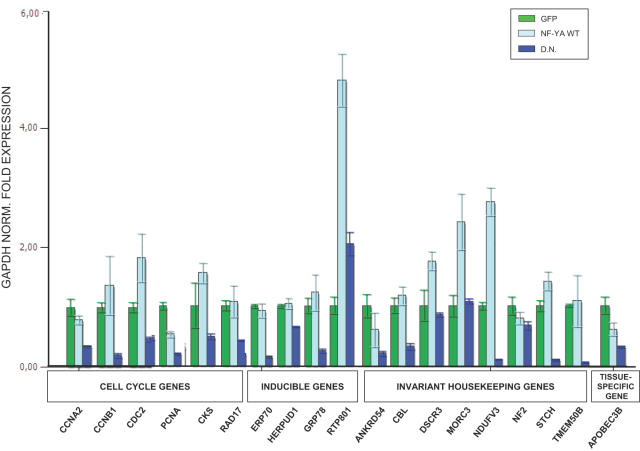
A Dominant Negative NF-YA affects CCAAT promoters activity. Quantitative RT-PCR analysis of growing HCT116 cells infected with control GFP, wtNF-YA and YAm29 (YA-DN) dominant negative Adenoviruses; the expression of 19 genes was evaluated after normalization for GAPDH levels. The genes were selected according to the expression patterns indicated. Five genes –ccna2, ccnB1, cdc2, grp78 and herpud1- are *bona fide* NF-Y targets, representing the positive control of the experiments. All other transcription units are derived from the ChIP on chip analysis on Chromosome 20, 21, 22 [24].

To correlate binding of NF-Y to gene expression, and the presence of active histone marks, we infected human HCT116 cells with an NF-YA dominant negative adenovirus (Ad-YA-DN) containing a mutation in the DBD subdomain (YAm29): the mutant protein associates with the histone fold dimer and renders the trimer incapable of CCAAT association [33 and References therein]. As controls, we infected cells with wt Ad-NF-YA and with Ad-GFP. The data were first normalized for GAPDH expression and Fig. 1 shows quantitative Real Time RT-PCR expression analysis of 19 NF-Y targets, in which binding was associated with H3K4Me3 in ChIP on chip experiments [24]; essentially, three patterns were observed: (i) in the majority, YA-DN treatment leads to decrease in expression of the genes, with little effect observed in the control GFP or Ad-NF-YA infections. (ii) For RTP801, DSCR3 and MORC3, Ad-NF-YA, but not YA-DN, infections lead to an increase in expression, compared to control GFP. (iii) For NF-2, the effect of the NF-Y viruses was inexistent, indicating that other TFs are more directly involved in transcription; this have been observed in other promoters and it is often a cell-type specific effect (R.M., unpublished).

### NF-Y is required for the establishment of active histone methyl marks on “open” promoters

**Figure 2:**
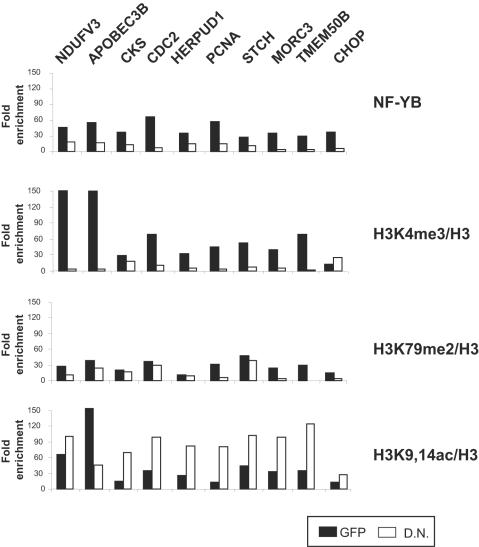
Removal of NF-Y leads to loss of H3 active methylation marks. HCT116 cell were infected as in Fig. 1, chromatin prepared and ChIP analysis performed with the indicated antibodies. Nine promoters dependent form NF-Y were evaluated in quantitative Real Time PCR analysis. Values are reported as fold enhancement over a control antibody –Flag- which was used in ChIPs. In parallel, we used an anti-H3 antibody to measure total H3 recovery. The values are normalized for the amount of H3 immunorecipitated in each point. Non normalized data are in Figure S1.

In the same experimental setting, we analyzed H3K4me3, H3K79me2 and H3K9-14ac by ChIP. Nine promoter locations of the genes analyzed above were controlled by Q-PCR. Initial evaluation of the data between chromatin of cells infected with Ad-GFP and wt Ad-NF-YA yielded similar results (See below), so we pursued analysis by comparing GFP and YA-DN. NF-Y binding was controlled with anti-YB antibodies, reasoning that for the YA-DN to be effective, efficient removal of one of the histone-like subunits should be monitored. With respect to the anti-Flag control antibody, binding was indeed severely affected -2 to 10-fold- on all promoters tested, although some residual binding was still observed (Fig. 2). ChIPs with histone marks were performed in parallel and evaluated after normalization for the total recoveries of histone H3, as measured with an antibody directed against non modified H3. The plotted data indicate a decrease of both H3K4me3 and H3K79me2 that paralleled almost perfectly that of NF-YB. The only exception was Chop, an inducible ER-stress gene, which is expressed at very low levels: indeed, it has a low level of H3K79me2, and H3K4me3 varies little. Interestingly, H3K9-14Ac behaved differently in YA-DN infected cells: in most promoters, there was a 2-fold increase, and only on APOBEC3B we observed a decrease. The raw data, non normalized by H3 recovery, are shown in Figure S1.

The decrease in H3K4me3 and H3K79me2 on CCAAT promoters could in theory be due to a generalized down-regulation of these marks in Ad-YA-DN-infected cells; similarly, the disappearance of NF-YB from promoters could be the result of inhibition of NF-YB mRNA synthesis by promoter interference of the YA-DN. We therefore assessed global levels of NF-Y subunits and histone modifications, by checking extracts of infected HCT116 in Western blots. Figure S2 shows the expected overexpression of NF-YA and YA-DN proteins and a small increase of NF-YC and NF-YB in YA-DN-treated cells. The levels of histones and H3 modifications were essentially unchanged, bar a slight decrease in H3K79me2 with YA-DN.

**Figure 3:**
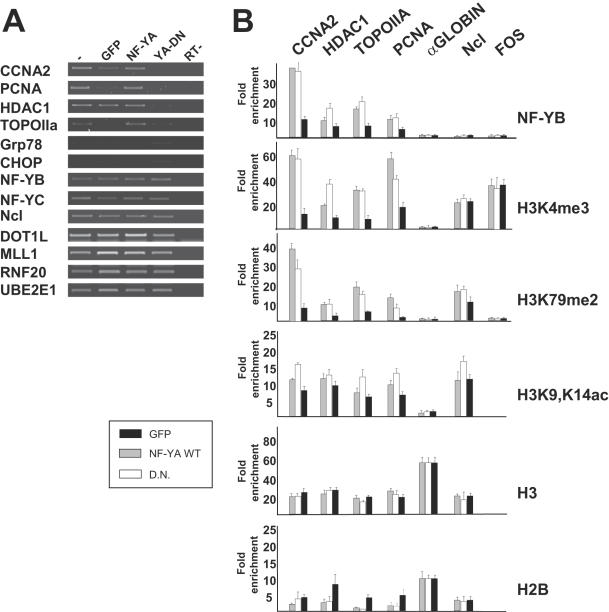
Effect of NF-Y removal in NIH3T3 cells. A. RT-PCR analysis of growing NIH-3T3 cells infected with control GFP, wtNF-YA and YAm29 (YA-DN) dominant negative Adenoviruses; NF-Y-dependent Cyclin A2, Pcna, Hdac1 and Topoisomerase IIα, ER inducible genes Grp78 and Chop, NF-Y independent nf-yb, nf-yc and nucleolin and histone modifying complex genes Mll1, Dot1l, Rnf20 and Ube2e1 are analyzed. B. ChIP analysis of NIH3T3 infected with GFP (Black bars), wtNF-YA (Grey bars) andYAm29 (White bars) viruses, with the indicated antibodies on the right. The promoter regions of genes listed on top of the figure were amplified. Values are measured as fold of enrichment over a Flag control antibody in semi-quantititaive PCR analysis [32].

We wished to extend our observations to another cellular context, the mouse non transformed fibroblasts NIH-3T3. Fig. 3A shows semi-quantitative RT-PCR analysis of four NF-Y target genes, *Ccna2*, *Pcna*, *Hdac1* and *Topo II α*: as expected, YA-DN treatment leads to decrease in expression of the genes, with little effect observed in the control GFP or Ad-NF-YA infections. The ER-stress inducible *Chop* and *Grp78* were essentially not expressed in the conditions used here. The expression levels of control, NF-Y-independent genes such as the *Nf-yb*, *Nf-yc* and *nucleolin* were not changed. We also checked expression of genes that impact on the H2B-Ub-H3K79-H3K4 pathway, *Rnf20*, *Ube2e1*, *Dot1l* and *Mll1*: none were altered by the YA-DN (Fig. 3). We then proceeded with ChIP analysis and found that YA-DN leads to a 3–6-fold reduction in NF-Y binding *in vivo*. A corresponding decrease in H3K4me3 and H3K79me2 was evident in all CCAAT promoters with YA-DN, but not with GFP nor NF-YA. On the other hand, H3K9-14ac was much less affected and only on H3 levels were not significantly changed. In this context, we also evaluated the levels of un modified histone H2B: interestingly, there is an increased on *Hdac1*, *Topo II a* and *Pcna* promoters, which parallel the decrease in NF-Y binding. On the other hand, the CCAAT *α-globin* promoter, which is inactive in NIH3T3, showed no active modifications and high H3-H2B levels; furthermore, the active CCAAT-less *nucleolin* and *fos* genes were negative for NF-Y, and the expected high levels of active marks were unaffected by the YA-DN.

We extended these observations to regions away from promoters. NF-Y was bound to the core promoter of all CCAAT genes analyzed and absent in the YA-DN condition (Figure S3). A decrease of H3K4me3 and H3K79me2 was visible in the same location after YA-DN infection, together with an increase of H2B, particularly sharp on *Chop*. Total H3 recovery was similar in all conditions. The levels of histone modifications were lower on upstream regions -1 Kb- and largely unaffected by YA-DN, whereas transcribed regions -+1 Kb- were modified, particularly H3K79me2 on *CyclinA2* and *Pcna*. This is consistent with evidence that this mark closely correlates with a transcribing RNA Polymerase II. We also probed extracts of infected NIH-3T3 by Western blots with the antibodies used for ChIPs, and the results were similar to those obtained with HCT116: only H3K79me2 was slightly reduced (Figure S2, Right Panels). Taken together, these data indicate that the binding of NF-Y to promoters of active, or poised genes is a crucial signal for the positioning methyl histone marks typical of active chromatin.

### NF-Y elimination prevents recruitment of the MLL complex

**Figure 4:**
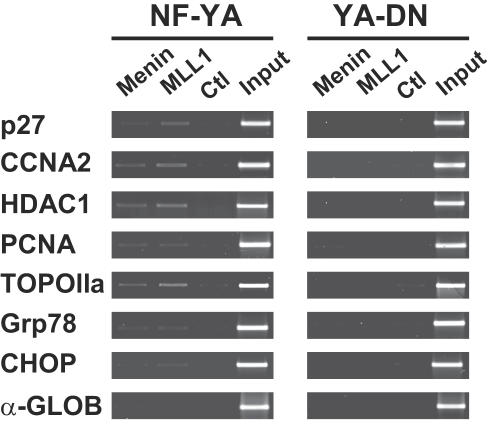
The MLL complex is not recruited on active promoters upon NF-Y removal. ChIP analysis of Menin and Mll1 on CCAAT promoters in NIH-3T3 cells infected with Ad wt NF-YA and YAm29 (YA-DN). The amplified promoter regions are indicated on the left of each PCR panel. p27 and α-globin are the positive and the negative controls, respectively.

Methylations of H3K4 and H3K79 are exerted by the MLL and Dot complexes, respectively [34], [35]. The data shown above would be consistent with either a lack of recruitment, or recruitment of inactive complexes that would be incapable of modifying H3 around promoter regions. We checked by ChIP whether elimination of NF-Y binding affects recruitment of MLL on CCAAT promoters. Figure 4 shows that while MLL proteins -MLL1 and Menin- are enriched over the Flag control on *Ccna2*, *Hdac1*, *Pcna*, *Topo II a* and, partially, *Chop* and *Grp78* in Ad-NF-YA infected cells (Left Panels), YA-DN largely prevents the association of both MLL proteins (Right Panels). As a positive control, we used *p27*, a bona fide target of MLL [36], which is also a known CCAAT promoter [37]: enrichments were similar in control conditions (YA), but were significantly reduced in the YA-DN infected cells. The silent *α-globin*, instead, was negative in both conditions. We conclude that removal of NF-Y from promoters prevents recruitment of key components of the MLL complex.

### NF-Y is required for H4K20me3 on many repressed promoters

**Figure 5:**
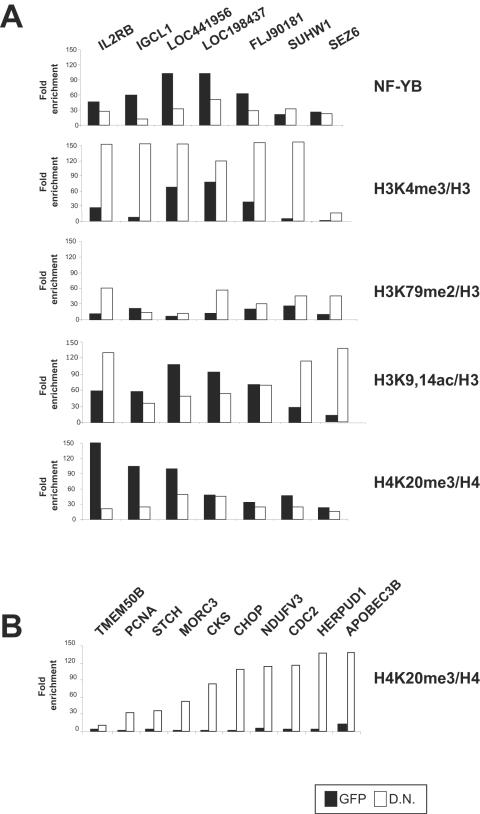
Removal of NF-Y leads to loss of H4K20me3 from inactive promoters and deposition of active methylation marks. A. HCT116 cells were infected with Ad-GFP and Ad-YA-DN, and chromatin analyzed by ChIP with the indicated antibodies. Seven promoters bound by NF-Y and H4K20me3 in ChIP on chip analysis [24] were evaluated in quantitative Real Time PCR analysis. Values are reported as fold enhancement over the control Flag antibody. In parallel, we used an anti-H3 and H4 antibodies to measure total histones recovery. The values are normalized for the amount of H3 and H4 immunorecipitated in each point. B. The H4K20me3 ChIPs were assayed for amplicons for promoters activated by NF-Y, after infections with Ad-GFP and Ad-YA-DN.

In addition to active marks, there are negative methyl modifications, such as H4K20me3. We recently reported the widespread presence on inactive CCAAT promoters bound by NF-Y in ChIP on chip experiments [24]. We therefore wondered whether NF-Y removal would alter this mark from these negatively regulated locations. We performed ChIPs in HCT116 cells and the data normalized for the amounts of H3 and H4 recovered. Five of seven targets showed a decrease in NF-Y binding and 3 in H4K20me3 (Fig. 5A); in parallel, H3K4me3, which showed only marginal enrichments in the GFP control, was substantially increased on five targets after Ad-YA-DN infections. Two promoters showed lesser change: LOC441956 and LOC198437. H3K79me2 was generally positively affected. H3K9-14Ac levels were high and an increase was observed on three genes -IL2RB, SUHW1 and SEZ6- which also show an increase in H3K4me3. In summary, the promoters in which the decrease in NF-YB association was more pronounced were also those in which the switch between positive and negative methyl marks are more prominent. To control for the specificity of these phenomena, we amplified the targets positively regulated by NF-Y described in Fig. 2: as shown in Fig. 5B, H4K20me3 was dramatically increased on essentially all targets, from background to 50- to 100-fold enrichments, after removal of NF-Y. This is in agreement with the decrease in positive methyl marks (Fig. 2) and repression of transcription (Fig. 1); note that Suhw1, which shows no change in the histone methyl patterns, is also invariant in the -low- transcription rates after NF-Y removal [24]. Therefore, we conclude that NF-Y controls a switch in methyl marks deposition and a dramatic change in promoter functions.

## Discussion

NF-Y is a sequence-specific histone-like transcription factor, whose binding location is usually near the transcriptional start sites. In this report, we find that DNA interactions are not affected by the presence of positive H3 methyl modifications. A *premiere* among transcription factors, NF-Y removal alters substantially the histone methylation patterns leading to important functional consequences.

### NF-Y in the build-up of positive methyl marks

It is clear that specific histone modifications are associated with “active” or “inactive” chromatin. Among active marks, genetic and biochemical experiments suggest a step-wise deposition of modifications along the promoter and transcribed regions of a gene. In general, a relevant question is what determines the location of these marks on genomes. It has long been known that the binding of transcription factors and cofactors to promoters are hallmarks of expression, by signalling to the Pol II transcription machinery the positional coordinates. It would seem logical, therefore, to postulate that positive histone marks are positioned according to a code determined by the sequence-specific TFs. Reassuringly, several studies have confirmed that TFs binding indeed correlates with the presence of “active” marks and with expression. In a detailed correlative analysis between histone marks and MYC binding *in vivo*, it was established that this TF is always associated with a specific context of H3K4me3 and H3K79me2. However, MYC removal -and comparison between myc^+/+^ and myc^−/−^ cells- leaves this pattern intact, while ablating H4 acetylations [19]. The conclusion was that MYC actually requires certain modifications to bind to the target E box and that binding is a signal for further modifications. Similarly, we and others found high levels of NF-Y binding and H3K4 and H3K79 methylations at poised promoters before induction [31], [32], begging for the question of which comes in first.

Surprisingly, in genome-wide analysis of NF-Y and H3K4me3 sites with tiling arrays, we find that NF-Y *loci* can be divided in two distinct groups: (i) an expected large one overlapping with H3K4me3 and correlating with expression; (ii) a second group, devoid of H3K4me3, on silent genes [24]. This suggested that the presence of this mark is not a pre-requisite for NF-Y binding. Rather, we find here that NF-Y binding is required for recruitment of key components of the MLL methylating complex –MLL1 and Menin- and for deposition of H3K4me3 and H3K79me2. It is apparent, therefore, that at least two classes of TFs exist: one which acts early in promoter identification and predisposes the recruitment of specific methylating complexes; a second class, *a la* MYC, which takes advantage of the “stage” set up by modifications set up by NF-Y (and the likes). This interpretation is entirely consistent with biochemical and functional experiments gathered in several inducible systems in which NF-Y, itself a poor “activator”, cooperates in recruiting neighbouring inducible TFs, such as HSF1 [38], SREBP1 [39], ATF6 [40]. Invariably, NF-Y binding precedes and is required for TF and coactivator positioning and transcriptional activation.

### NF-Y and H4K20me3

A role of NF-Y in active mechanisms of repression is emerging [33 and References therein] and recent experiments in *C. elegans* have genetically linked ceNF-Y to Polycomb repression through ESC/E(Z) [41]. It was therefore not completely unexpected to find that the NF-Y dependence of positive marks on active promoters is mirrored, on repressed genes, by the behaviour of H4K20me3. We have specifically analyzed this mark because it was the most consistently counter-correlative to H3K4me3 in the cohort of *loci* derived from our ChIP on chip experiments. As for the other two major negative methyl marks, we could not find any enrichment of H3K9me3, and only a subset of H3K4me3^−^ sites were H3K27me3^+^. H4K20 tri-methylation is performed by the Suv4-20 Set domain methylase and is apparently associated with heterochromatin formation [14]. NF-Y appears to be important to anchor this negative mark on many, but not all promoters: we note that the results, in this set, are more variegated with respect to positive marks: LOC441956, IL2RB, IGCL1 show a parallel decrease of NF-YB and H4K20me3, LOC198537 and FLJ90181 of the former by marginally of the latter, SUHW11 of the latter, but not the former, SEZ6 of neither. There is an important issue that needs to be underlined when considering the lack of NF-YB removal in ChIPs: we are measuring NF-Y binding with an antibody against NF-YB, under conditions in which the dominant negative is NF-YA-based; the lack of removal of the histone-fold dimer could be due to additional interactions with other TFs, independently from the CCAAT box. The NF-YB-NF-YC dimer, indeed, has several residues in the L1 and L2 that could contact DNA *a la* H2A-H2B [27 and References therein]. SUHW1 and SEZ6, which do respond to Ad YA-DN infections by switching histones marks, but maintaining NF-YB association, might uncover a specific role of the NF-YA subunit in this switch, uncoupled to the dimer. In addition, there is an excess of NF-YB-NF-YC over NF-YA -2–3-fold on average (M. Ceribelli, in preparation)- and thus there might be sites occupied by the histone-fold dimer, but not by NF-YA: these would be predicted to be oblivious of the NF-YA dominant negative, as used here. It will be essential to perform genome-wide analysis of NF-YA and specific inactivation of the histone fold dimer to establish clarify these points.

### NF-Y as a variant H2B-H2A?

Monoubiquitination of H2B at Lysine 123 -K120 in humans- by the E2 conjugating RAD6 and the E3 ubiquitin ligase BRE1 is an early event in the establishment of a chromatin environment conducive of transcription [Reviewed in Refs. 34], [42]. H3K79me2 is methylated in an H2B-Ub-dependent manner in budding yeast [9], [11], and the role of hBRE1 -RNF20/40- in methylation of H3K4 and H3K79 has been established *in vitro* and *in vivo* [18], [43]. H2A is also monoubiquitinated by a Ring protein of the Polycomb complex, and the functional significance is opposite to H2B-Ub: repression [44]. The crystal structure of NF-YB/NF-YC has been solved and the H2A-H2B histone-like nature clearly emerged [27]. In particular, H2A-H2B basic residues in L1 and L2 loops that contact DNA within the nucleosome [45] are conserved and required for NF-Y-CCAAT interactions [46]. H2B modifications are mostly in tails, but some are within the histone-fold, notably K43me and K85ac, both contacting the phosphate backbone of DNA [47]. Interestingly, these two Lysines are conserved in NF-YB and required for DNA-binding [46]. Furthermore, the NF-YB Lysine corresponding to H2B K85 -K107- is acetylated by p300 (G. Caretti, R.M., unpublished). There is currently no data about NF-Y ubiquitination, but the molecular basis for modifications are present, both in NF-YB (K135, K140, K146, K149 in the α-C) and NF-YC (K124 and K127 also in α-C). Clearly, this is an area that will have to be investigated thoroughly in the future.

The switch between positive and negative marks detailed here leads us to speculate that NF-Y might represent a specialized version of the H2A-Ub/H2B-Ub system, one that would have a strict sequence-specificity and affinity for core promoters. In an elegant study on the rules determining global nucleosome positioning in yeast, Segal et al. found low nucleosome occupancy at TSS and examined frequencies of TFs for the occurrence of nucleosome occupancy: the yeast NF-Y homologues HAP2/3/5 came on top of the ranking for binding site accessibility by intrinsic nucleosome positioning [48]. Thus, CCAAT boxes would be specifically left open for binding in core promoters. We propose a scenario whereby NF-Y is a “variant” sequence-specific histone, more distantly related with respect to H2A.Z and H3.3 [49], that marks transcription units containing the CCAAT recognition sequence. Much biochemical and genetic work lies ahead to establish whether there is indeed an “NF-Y code” of modifications that funnels histone modifying machines to promoters and other regulatory areas of the genome and thus regulating local chromatin accessibility.

## Materials and Methods

### Cells and Infections

NIH3T3 cells were grown in DMEM supplemented with 10% Foetal Calf serum (FCS), 1% antibiotics (penicillin and streptomycin) and L-glutamine in 5% CO2. HCT116/p53^−/−^ cells were grown in McCOY'S medium. Infections with Ad-YAm29, Ad-YAwt and Ad-GFP adenoviruses were carried out as described previously (Imbriano et al, 2005).

### Chromatin Immunoprecipitation

Standard chromatin preparation and ChIP assays were performed as previously described (Donati et al, 2006) with minor modifications. Briefly, crosslinked chromatin from 5×10^6^ HCT116/p53^−/−^ and NIH3T3 cells, was sonicated to fragments length of approximately 1/1.5 Kb and immunoprecipitated with 3–5 µg of each antibodies.

Antibodies: NF-YB (Diagenode, Belgium), H3K4me3 (Abcam 8580, UK), H3K79me2 (Abcam 3594), H3K9-14Ac (Upstate 06599), H4K20me3 (Abcam 9053-100), unmodified H3 (Abcam 1791), unmodified H2B (Abcam 1790), unmodified H4 (Abcam 7311-200), MLL1 (Gift of Eli Canaani), Menin (BL342 Bethyl, USA) and Flag (Sigma, USA). The ChIP-PCR primers used for these experiments are reported in Table S1.

### Real time PCR

Quantitative Real Time PCR was performed using SYBR green IQ reagent (Biorad) in the iCycler machine (Biorad). Primers were designed to amplify genomic regions of 100–150 bp size and are listed in Table S1. The relative sample enrichment was calculated with the following formula: 2^ΔCtx^−2^ΔCtb^, where ΔCt x = Ct input-Ct sample and ΔCt b = Ct input-Ct control Ab. Data from semi-quantitative PCRs were analyzed as in Ref. 32.

### Western blot analyses

Total Extracts (50 µg) of HCT116/p53−/− and NIH3T3 infected with Adenoviruses were prepared and Western blots were performed according to standard procedures, with the indicated primary antibodies and a peroxidase conjugated secondary antibody (Amersham).

## Supporting Information

Figure S1 (0.05 MB PPT)

Figure S2 (3.54 MB PPT)

Figure S3 (0.02 MB PDF)

Table S1 (0.03 MB XLS)
