# Correction: An NF-Y-Dependent Switch of Positive and Negative Histone Methyl Marks on CCAAT Promoters

**DOI:** 10.1371/journal.pone.0102282

**Published:** 2014-07-17

**Authors:** The PLOS ONE Staff

There are errors in Figures 3, 4 and Supporting Information Figure S2 which the authors wish to correct: the panel of CHOP in the published Figure 3 duplicates the panel of Grp78; the right panel of HDAC1 in the published Figure 4 duplicates the panel of p27; the gene names characters are incorrectly capitalized; there are errors in some of the data for the control experiments displayed in the HCT116 (left) panels of Supplementary Figure 2. These errors do not affect the conclusions reported in the article. While some of the errors may be related to the processing of the files during the production process, the authors consulted the Commission of Scientific Integrity appointed by the Department of Biosciences, University of Milan in relation to the data from this study. The Commission supports the publication of a Correction. The authors apologize for these errors and provide a revised version for each of the figures above with the original, correct panels. The raw blots for each figure are also available via this Correction.

Please see the correct Figure 3 here.

**Figure 3:**
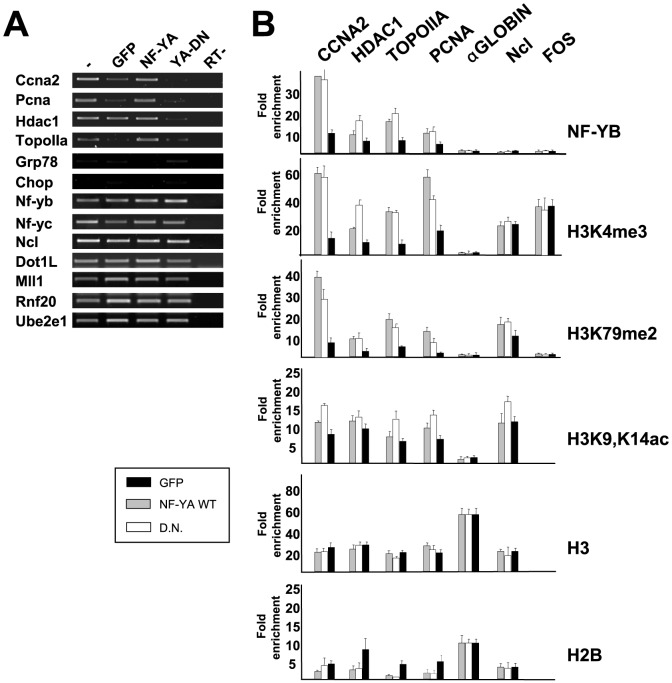
Effect of NF-Y removal in NIH3T3 cells. A. RT-PCR analysis of growing NIH-3T3 cells infected with control GFP, wtNF-YA and YAm29 (YA-DN) dominant negative Adenoviruses; NF-Y-dependent Cyclin A2, Pcna, Hdac1 and Topoisomerase IIα, ER inducible genes Grp78 and Chop, NF-Y independent nf-yb, nf-yc and nucleolin and histone modifying complex genes Mll1, Dot1l, Rnf20 and Ube2e1 are analyzed. B. ChIP analysis of NIH3T3 infected with GFP (Black bars), wtNF-YA (Grey bars) andYAm29 (White bars) viruses, with the indicated antibodies on the right. The promoter regions of genes listed on top of the figure were amplified. Values are measured as fold of enrichment over a Flag control antibody in semi-quantititaive PCR analysis [32].

Please see the correct Figure 4 here.

**Figure 4:**
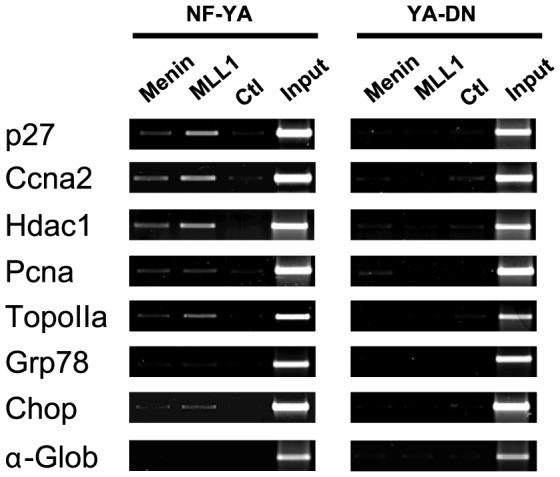
The MLL complex is not recruited on active promoters upon NF-Y removal. ChIP analysis of Menin and Mll1 on CCAAT promoters in NIH-3T3 cells infected with Ad wt NF-YA and YAm29 (YA-DN). The amplified promoter regions are indicated on the left of each PCR panel. p27 and á-globin are the positive and the negative controls, respectively. Please see the correct Supporting Information Figure S2 here.

## 

Figure S2 Western blot analysis of extracts of cells infected with GFP, Ad-NF-YA and Ad-YA-DN, with antibodies against NF-YA, NF-YB, NF-YC, and the indicated histone modifications. Left Panels, HCT116; Right Panels, NIH3T3. YA l and YA s refer to the two splicing isoforms of NF-YA: note that HCT116 mainly express the short isoform and that Ad vectors express the long isoform.
(PDF)

Raw Blots File S1 Please see the raw blots 1, 2 and 3 here.
(ZIP)
