# Supplementary material for: An NF-Y-Dependent Switch of Positive and Negative Histone Methyl Marks on CCAAT Promoters
Source: PLoS One. 2008 Apr 30;3(4):e2066. doi: 10.1371/journal.pone.0002066 (PMC2312324; doi:10.1371/journal.pone.0002066)
Supplement: Figure S1 — (0.05 MB PPT) [file pone.0002066.s001.ppt]

## Slide 1
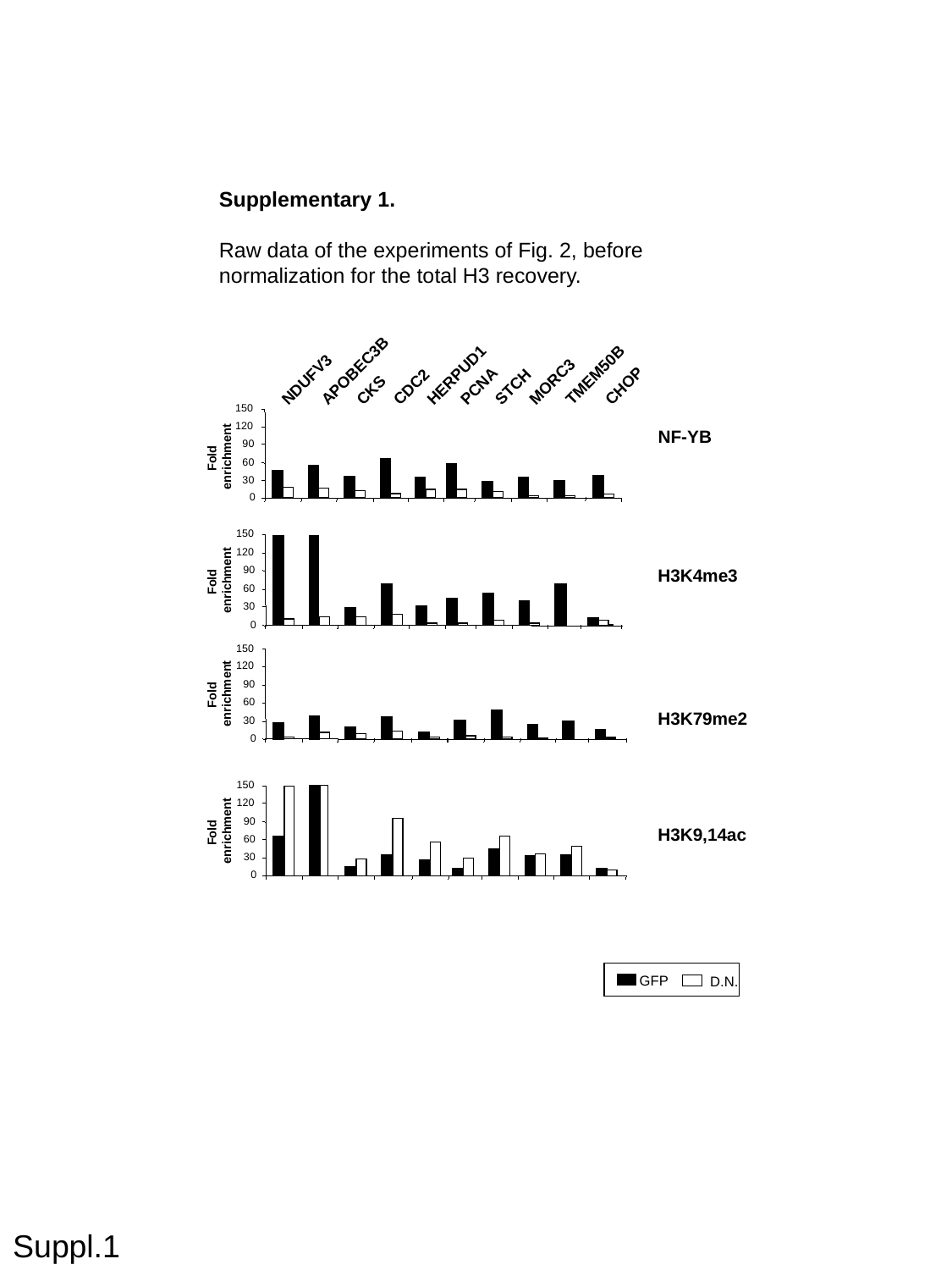

Supplementary 1.
Raw data of the experiments of Fig. 2, before normalization for the total H3 recovery.
APOBEC3B
HERPUD1
TMEM50B
NDUFV3
MORC3
CHOP
PCNA
CDC2
STCH
CKS
150
120
90
60
30
0
NF-YB
Fold
 enrichment
150
120
Fold
enrichment
90
H3K4me3
60
30
0
150
120
90
60
30
0
Fold
enrichment
H3K79me2
150
120
Fold
 enrichment
90
H3K9,14ac
60
30
0
GFP
D.N.
Suppl.1
