# Supplementary material for: An NF-Y-Dependent Switch of Positive and Negative Histone Methyl Marks on CCAAT Promoters
Source: PLoS One. 2008 Apr 30;3(4):e2066. doi: 10.1371/journal.pone.0002066 (PMC2312324; doi:10.1371/journal.pone.0002066)
Supplement: Figure S2 — (3.54 MB PPT) [file pone.0002066.s002.ppt]

## Slide 1
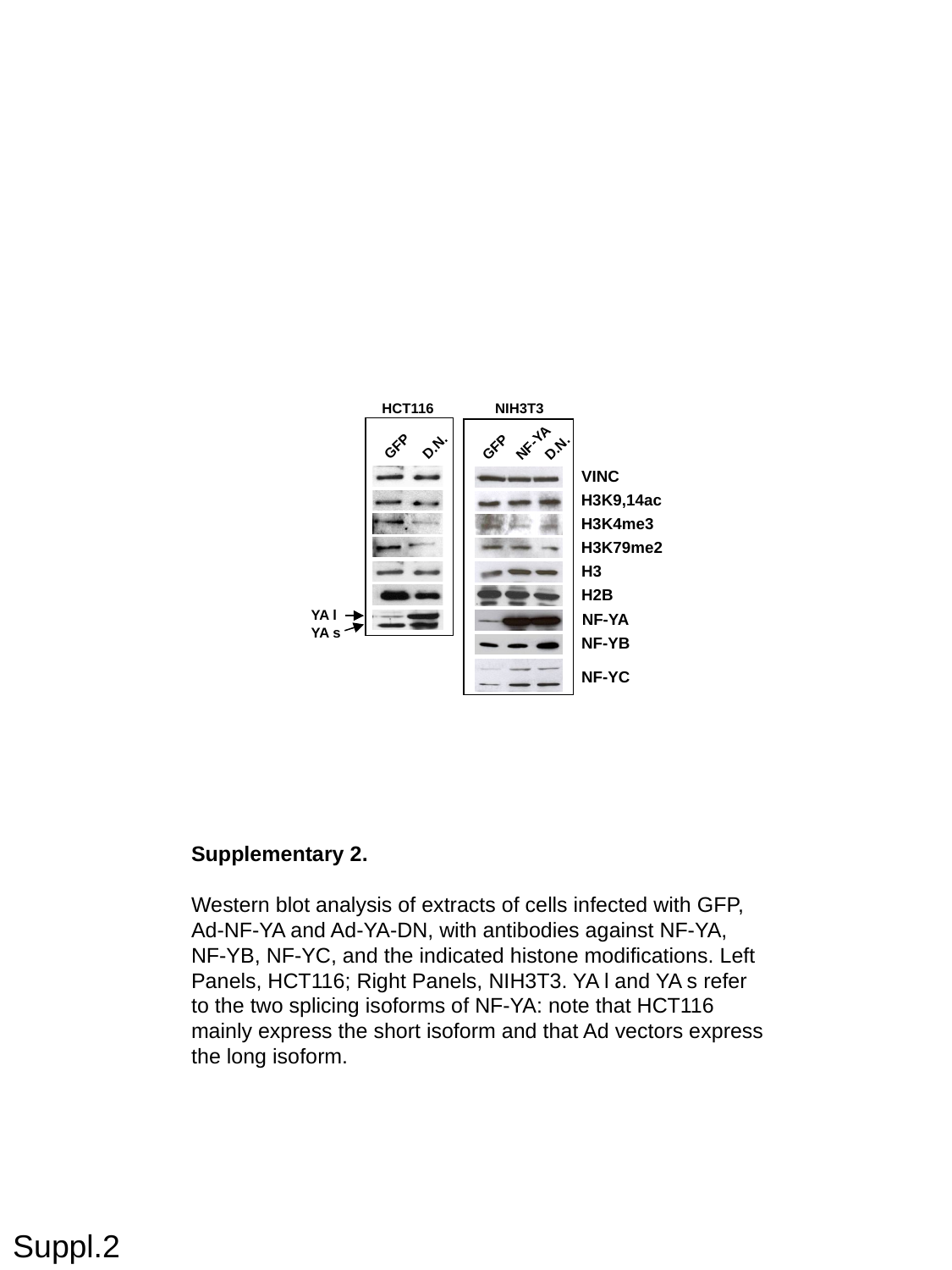

HCT116
NIH3T3
NF-YA
GFP
D.N.
GFP
D.N.
VINC
H3K9,14ac
H3K4me3
H3K79me2
H3
H2B
YA l
NF-YA
YA s
NF-YB
NF-YC
Supplementary 2.
Western blot analysis of extracts of cells infected with GFP, Ad-NF-YA and Ad-YA-DN, with antibodies against NF-YA, NF-YB, NF-YC, and the indicated histone modifications. Left Panels, HCT116; Right Panels, NIH3T3. YA l and YA s refer to the two splicing isoforms of NF-YA: note that HCT116 mainly express the short isoform and that Ad vectors express the long isoform.
Suppl.2
