# Supplementary material for: An NF-Y-Dependent Switch of Positive and Negative Histone Methyl Marks on CCAAT Promoters
Source: PLoS One. 2008 Apr 30;3(4):e2066. doi: 10.1371/journal.pone.0002066 (PMC2312324; doi:10.1371/journal.pone.0002066)

Supplementary 3.

Loss of H3 active methylation marks is specific to promoters and transcribed regions. ChIP analysis of Grp78 and Chop upstream (-1kB), core promoter (TSS) and transcribed regions (+1kB); CyclinA2 (+1kB) and Pcna (+1.7kB) transcribed regions with growing NIH-3T3 infected with the indicated adenoviruses as in Fig. 3. The antibodies are listed on the right and fold enrichment was calculated as described for Fig. 3

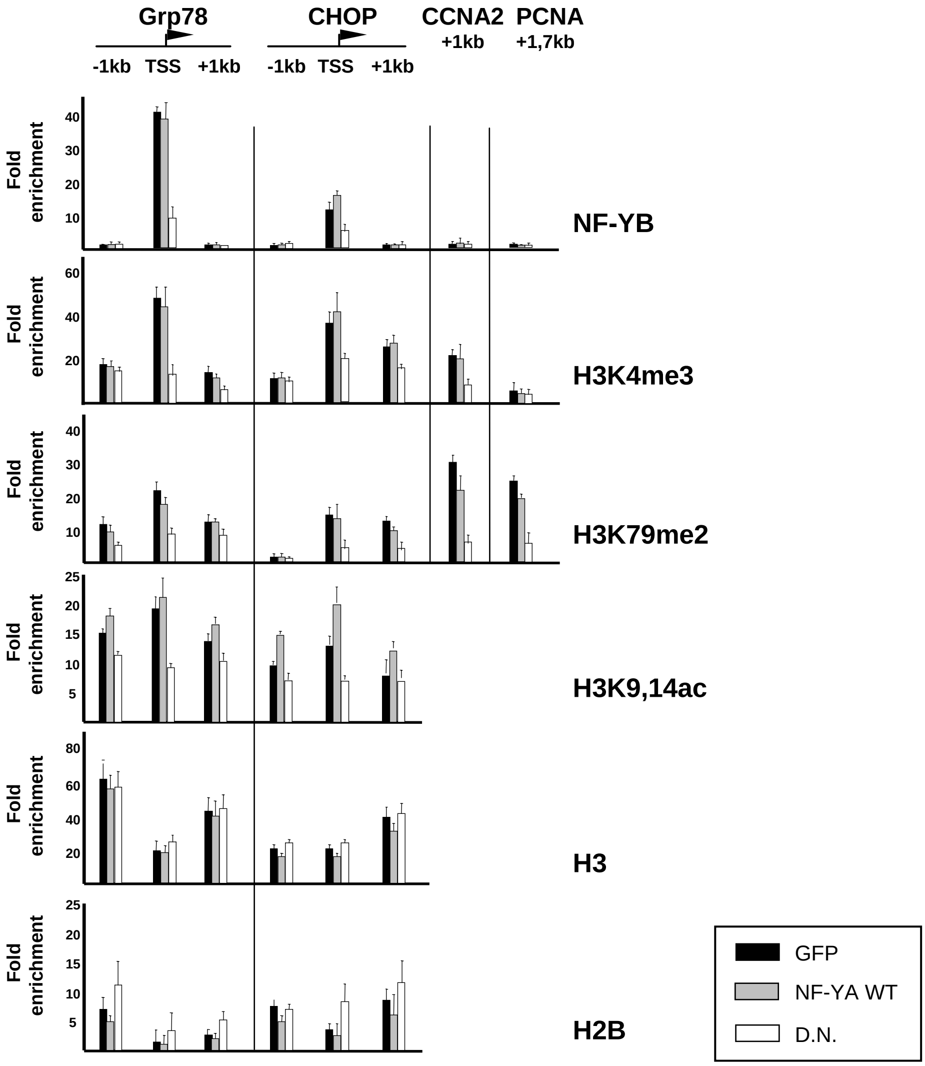

Supplement: Figure S3 — (0.02 MB PDF) [file pone.0002066.s003.pdf]
