# Supplementary material for: Correction: An NF-Y-Dependent Switch of Positive and Negative Histone Methyl Marks on CCAAT Promoters
Source: PLoS One. 2014 Jul 17;9(7):e102282. doi: 10.1371/journal.pone.0102282 (PMC4102492; doi:10.1371/journal.pone.0102282)
Supplement: Figure S2 — Western blot analysis of extracts of cells infected with GFP, Ad-NF-YA and Ad-YA-DN, with antibodies against NF-YA, NF-YB, NF-YC, and the indicated histone modifications. Left Panels, HCT116; Right Panels, NIH3T3. YA l and YA s refer to the two splicing isoforms of NF-YA: note that HCT116 mainly express the short isoform and that Ad vectors express the long isoform. (PDF) [file pone.0102282.s001.pdf]

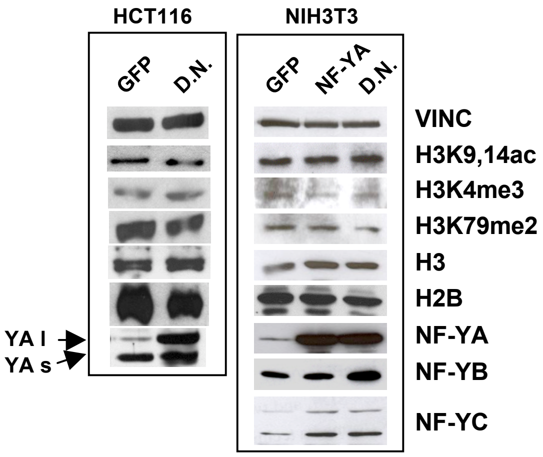

Supplementary 2.

Western blot analysis of extracts of cells infected with GFP, Ad-NF-YA and Ad-YA-DN, with antibodies against NF-YA, NF-YB, NF-YC, and the indicated histone modifications. Left Panels, HCT116; Right Panels, NIH3T3. YA l and YA s refer to the two splicing isoforms of NF-YA: note that HCT116 mainly express the short isoform and that Ad vectors express the long isoform.

Suppl.2
